# An assessment of extensive intraductal component as a risk factor for local recurrence after breast-conserving therapy.

**DOI:** 10.1038/bjc.1990.195

**Published:** 1990-06

**Authors:** J. Jacquemier, J. M. Kurtz, R. Amalric, H. Brandone, Y. Ayme, J. M. Spitalier

**Affiliations:** Department of Anatomic Pathology, Marseille Cancer Institute, France.

## Abstract

The influence of extensive intraductal component (EIC) on local recurrence risk was studied for 496 patients with stage I-II infiltrating ductal cancers treated by conservative surgery and irradiation. EIC was diagnosed in 65 of 231 (28%) premenopausal and 41 of 265 (15.5%) post-menopausal patients. Local recurrence risk was markedly increased in EIC+ patients (5-year actuarial risk 18% versus 8% without EIC, P less than 0.001), but this effect appeared limited to premenopausal patients. Local recurrence risk increased with increasing degree of EIC. EIC with more than 50% intraductal carcinoma was more prevalent in patients younger than 40, perhaps accounting to some degree for the higher local recurrence rates observed in younger patients. The presence of EIC had no influence on overall survival, on median time to local recurrence, or on short-term survival after local failure. The usefulness of EIC as a risk factor for local recurrence is discussed.


Br. J. Cancer (1990), 61, 873-876 © Macmillan Press Ltd., 1990

### An assessment of extensive intraductal component as a risk factor for local recurrence after breast-conserving therapy

J. Jacquemier[1], J.M. Kurtz[3], R. Amalric[2], H. Brandone[2], Y. Ayme[2] & J.-M. Spitalier[2]

[1]*Department of Anatomic Pathology, Marseille Cancer Institute;* [2]*Académie Méditerrannéenne d'Oncologie Clinique, Marseille, France; and* [3]*the Radiation Oncology Department, University Hospital, Petersgraben 4, CH-4031 Basle, Switzerland.*

**Summary** The influence of extensive intraductal component (EIC) on local recurrence risk was studied for 496 patients with stage I-II infiltrating ductal cancers treated by conservative surgery and irradiation. EIC was diagnosed in 65 of 231 (28%) premenopausal and 41 of 265 (15.5%) post-menopausal patients. Local recurrence risk was markedly increased in EIC+ patients (5-year actuarial risk 18% versus 8% without EIC, $P<0.001$), but this effect appeared limited to premenopausal patients. Local recurrence risk increased with increasing degree of EIC. EIC with more than 50% intraductal carcinoma was more prevalent in patients younger than 40, perhaps accounting to some degree for the higher local recurrence rates observed in younger patients. The presence of EIC had no influence on overall survival, on median time to local recurrence, or on short-term survival after local failure. The usefulness of EIC as a risk factor for local recurrence is discussed.

Extensive intraductal component (EIC), as defined by Schnitt *et al.* (1984), is said to be present when 25% or more of an invasive breast cancer consists of intraductal carcinoma, and ductal carcinoma *in situ* (DCIS) is also present outside the main tumour mass. Available evidence indicates that the presence of EIC correlates positively with the quantity of residual cancer remaining in the breast after conservative excision (Schnitt *et al.*, 1987; Holland *et al.*, 1990), and clinical studies from Harvard University suggest that tumours with EIC have a high risk of recurring in the breast, despite adequate radiotherapy (Recht *et al.*, 1986).

Although EIC has been viewed with scepticism by some authors (Fisher *et al.*, 1986; Calle *et al.*, 1986), it not clear whether or not an effort had been made by other investigators to reproduce the definitions used by the Harvard group. Evaluation of the clinical significance of EIC was one of the goals of a retrospective clinico-pathological study of the risk factors for local recurrence carried out at the Cancer Institute in Marseille, the overall results of which will appear elsewhere (Kurtz *et al.*, 1990). The purpose of the current paper is to examine more closely the potential influence of EIC on local recurrence risk, with a detailed comparison of our results with those of the Harvard group. In addition, the question of the age dependence of EIC will be addressed, as well as the possible influence of EIC on overall survival, time interval to recurrence, and survival after recurrence.

### Patients and methods

The study group consisted of 496 evaluable patients with clinical stages I-II (American Joint Committee, 1983) infiltrating ductal carcinomas who had breast-conserving surgery at the Cancer Institute in Marseille between January 1975 and December 1983. This formed the major part of a study including 587 cancers of all histological subtypes (Kurtz *et al.*, 1989). Briefly, primary treatment consisted of wide excision of the primary tumour, including dissection of the lower two axillary levels in 487 patients. Radiotherapy delivered 50 Gy in 5 weeks to the breast and regional nodes on telecobalt, or its equivalent on telecesium, followed by supplemental irradiation of 20-25 Gy to the tumour bed using an electron beam. Selected patients also received adjuvant hormonal or chemotherapy with various agents.

Histological slides of all primary tumours were reviewed without prior knowledge of the treatment outcome. The 496 cases classified as infiltrating ductal carcinomas also included all cases of tubular carcinoma (World Health Organization, 1982), atypical medullary carcinoma (Rapin *et al.*, 1988), as well as invasive ductal carcinomas with predominant intraductal component (World Health Organization, 1982). The amount of intraductal cancer within the primary tumour was estimated as less than 25%, 25-50% or more than 50%, and outside the tumour mass as being either absent or present, as suggested by Schnitt *et al.* (1984). EIC was diagnosed when 25% or more of the tumour consisted of DCIS and DCIS was also present in the periphery.

Resection margins were reviewed retrospectively, using sections taken through the tumour, as well as separate sections from the periphery. Although the surgeon had performed what was considered a macroscopically complete excision, no intra-operative frozen section control of resection margins had been carried out, and the specimens had not been marked with India ink. In some cases, therefore, a confident assessment of microscopic resection margins could not be made, especially when peripheral sections were unavailable for review; in such instances, margins were classified as indeterminate.

Actuarial calculations were performed according to methods described by Kaplan and Meier (1958). Local recurrence included recurrent cancer in the parenchyma or skin of the treated breast, regardless of prior events. Overall survival included deaths from all causes. Differences between actuarial curves were tested for significance by the log rank test, and differences between proportions by the $\chi^2$ test (Peto *et al.*, 1977).

### Results

### *Correlation of EIC with local recurrence risk*

After a median follow-up of 71 months, recurrent cancer in the treated breast was observed in 61 of 496 (12%) patients. Local failure was very significantly associated with both the degree of DCIS within the primary tumour and the presence of intraductal cancer in its periphery (Table I). Local recurrence occurred in 22 of 114 (19%) tumours containing 25% or more DCIS, compared with 39 of 382 (10%) with lesser amounts ($P<0.01$). Similarly, failure in the breast was observed in 41 of 269 (15%) of tumours with DCIS in their periphery, compared with only 20 of 227 (9%) in tumours having no demonstrable intraductal cancer in breast tissue outside the invasive lesion ($P<0.05$). However, in only eight instances was extensive DCIS diagnosed within the tumour without some degree of intraductal carcinoma being identified in the periphery, and in none of these was a local

Correspondence: J.M. Kurtz.
Received 9 November 1989; and in revised form 16 January 1990.

recurrence observed. It is apparent from Table I that the high local recurrence risk is associated with the subgroup demonstrating both adverse features, corresponding to the definition of EIC proposed by the Harvard group (Boyages *et al.*, 1989). The 5-year actuarial local failure rate for tumours with and without EIC are 18% and 8%, respectively ($P<0.001$). The corresponding computer-generated curves for actuarial risk of local breast recurrence are shown in Figure 1 for each of the two groups.

*Correlation of EIC with type of local recurrence*

Compared with 390 patients without EIC, the 106 EIC+ patients had an increased incidence of operable failure near the primary tumour bed (16 of 106, 15%, versus 18 or 390, 4.6%, $P<0.001$), whereas there was no difference in the incidence of either operable recurrence elsewhere in the breast (3 of 106, 2.8%, versus 12 of 390, 3.1%) or of inoperable recurrence (3 of 106, 2.8%, versus 9 of 390, 2.3%).

*Importance of the degree of EIC*

The influence of the degree of EIC on local recurrence was investigated. EIC+ patients with more than 50% DCIS had significantly more local recurrences than those with 25–50% DCIS (Table II), whereas the local failure rate in the latter group was only slightly higher than in the EIC− group.

*Influence of EIC on survival*

Acturarial overall survival from time of initial therapy is shown in Figure 2 for both EIC+ and EIC− patients. Despite the marked differences in local recurrence between the two groups, the survival curves were identical, with a 10-year survival of 72% in each case.

**Table I** Dependence of local recurrence in the treated breast on the extent of intraductal cancer within the primary tumour and on the presence or absence of intraductal cancer outside the infiltrating tumour

| | *Crude breast recurrence rate DCIS adjacent to tumour* | |
|---|---|---|
| *DCIS within tumour* | *Present* | *Absent* |
| 25% or more | *22/106 (21%)* | 0/8 (0%) |
| Less than 25% | 19/163 (12%) | 20/219 (9%) |

The subgroup with extensive intraductal component (EIC) is in italics. DCIS = intraductal carcinoma.

**Figure 1** Actuarial probability of local recurrence in the treated breast, according to the presence or absence of extensive intraductal component (EIC). The numbers below the time axis indicate the number of breasts at risk for each interval.

**Table II** Dependence of crude local recurrence rate on the degree of intraductal component

| | *Crude breast recurrence rate* | |
|---|---|---|
| EIC− | 39/390 (10%) | $P<0.001$ |
| EIC+ | 22/106 (21%) | |
| with 25–50% DICS | 10/70 (14%) | $P<0.05$ |
| with >50% DCIS | 12/36 (33%) | |

DCIS = intraductal cancer. EIC = extensive intraductal component.

**Figure 2** Actuarial overall survival following initial treatment, according to the presence or absence of extensive intraductal component (EIC). The numbers below the time axis indicate the number of patients at risk for each interval. n.s. = not significant.

*Relation of EIC to resection margins*

Retrospective review of resection margins indicated that EIC+ patients more commonly had inadequate excision (24 of 106, 23%) than did EIC− patients (25 of 390, 6.5%, $P<0.001$). However, in the presence of EIC, local recurrence rate was high, regardless of whether resection margins were negative (5 of 27, 18.5%), positive (7 of 24, 29.2%) or indeterminate (10 of 55, 18.2%).

*Correlation of age with EIC*

EIC was found more frequently in premenopausal (65 of 321, 28%) than in post-menopausal patients (41 of 265, 15.5% $P<0.05$). The age distribution according to the degree of EIC is shown in Table III. The prevalence of EIC is similar in both older age groups. The higher frequency of EIC in younger patients reflects a significantly greater prevalence of 25–50% DCIS in patients age 40–49 and of the extreme form of EIC (more than 50% DCIS) in patients younger than 40.

**Table III** Dependence of the degree of extensive intraductal component on patient age for 496 patients with infiltrating ductal carcinomas

| | | *Prevalence (%)* | | |
|---|---|---|---|---|
| *Age* | *(n)* | *>50% DCIS* | *25–50% DCIS* | *Total EIC+* |
| <40 | (62) | *11 (18%)* | 7 (11%) | *18 (29%)* |
| 40–49 | (147) | 11 (8%) | *33 (22%)* | *44 (30%)* |
| 50–59 | (161) | 9 (6%) | 18 (11%) | 27 (17%) |
| ⩾60 | (126) | 5 (4%) | 12 (9%) | 17 (13%) |
| *P* | | 0.001 | 0.001 | 0.025 |

The italicised values in each column are significantly different from those not italicised ($\chi^2$ test). DCIS = intraductal carcinoma within primary tumour.

The influence of EIC on local recurrence risk according to menopausal status is presented in Table IV. The importance of EIC as a risk factor appears confined to premenopausal patients. Post-menopausal patients with EIC have an identical local failure rate to patients without EIC. For EIC + patients, the difference in local failure rate between pre- and post-menopausal patients was of borderline statistical significance ($P<0.09$).

*Influence of EIC on the course of recurrent patients*

For 22 EIC + patients and 39 EIC − patients suffering local failure, both the median time interval between initial treatment and local recurrence and the short-term survival after recurrence were similar for both groups. Local failure for EIC + patients occurred after a median interval of 29 months (range 11–118 months), for EIC − cases after a median interval of 29.5 months (range 8–98 months). Three-year overall survival after local failure was 51.4% for EIC + and 55.5% for EIC − patients (median follow-up after failure 26 months).

### Discussion

The current study confirms that the presence of EIC correlates significantly with the probability of local recurrence after conservative surgery and radiotherapy. However, this analysis points out certain characteristics which may limit the degree of usefulness of EIC as a risk factor, perhaps explaining why its importance has not been uniformly recognised by other groups examining more limited case material (Fisher *et al.*, 1986; Calle *et al.*, 1986). These problematic aspects include the reproducibility of the diagnosis of EIC, the possible interactions of EIC with resection margins in determining risk, and the effect of age both on the incidence of EIC and the probability of local recurrence.

Studies of re-excision after previous biopsy (Schnitt *et al.*, 1987) as well as of serial subgross sectioning of whole breast specimens following simulated tumorectomy (Holland *et al.*, 1990) strongly suggest that EIC is an important marker of residual cancer after local tumour resection. Based on these data, the Harvard group has advanced the hypothesis that extensive residual intraductal cancer in the vicinity of the excised primary lesion is responsible for the high local failure rate associated with EIC (Boyages *et al.*, 1989). A direct assessment of the quantity of DCIS outside the primary tumour mass would most likely provide the best marker for residual intraductal cancer beyond the margins of excision, but the rim of macroscopically normal breast tissue available for pathological examination is generally too narrow to allow a consistently reliable evaluation of this sort. Apparently the extent of DCIS within the tumour provides a valid indirect measure of residual cancer, provided that DCIS can in fact be documented in the periphery as well (Table I).

Overall, our results correspond rather closely to those of the Harvard group, with 5-year actuarial local failure rates with and without EIC of 18% versus 8% for the Marseille patients and 25% versus 5% for the Boston patients. However, whereas the majority of local failures (36 of 53, 68%) in the patients reported by Boyages *et al.* (1989) were associated with EIC, only 22 of the 61 recurrences in our series could be attributed to this risk factor. Thus, significantly more recurrences were noted in the Marseille than in the Boston series for patients having less than 25% DCIS within the tumour (39 of 382, or 10.2% versus 16 of 279, or 5.7%, $P<0.01$). In this sense, our data support, at least to a limited extent, the clinical usefulness of the concept of EIC as defined by Schnitt *et al.* (1987) but suggest that other explanations should be sought for the substantial number of local failures not accounted for by this single risk factor. In addition, we have shown that the extreme form of EIC, with more than 50% DCIS within the tumour, appears to be associated with a particularly high risk of local relapse (Table II). As in the Boston series, EIC predicts uniquely for a higher likelihood of operable recurrence in the vicinity of the primary tumour, while the risk of recurrence elsewhere in the breast or of inoperable recurrence seem unaffected by EIC.

It is interesting to note that the diagnosis of EIC was made significantly less often in our series than in the Boston patients (106 of 496, 21%, versus 143 of 445, 32%, $P<0.001$). This may reflect a true difference in the prevalence of EIC between breast cancer patients in Massachusetts and those in the south of France, or may be related to differences in age distribution. It is likely, however, that our ability to diagnose EIC had been hindered to some degree by the relatively small number of histological sections available for review in this study (mean number 4.1). In addition, the semiquantitative assessment required to diagnose EIC is likely to be carried out in a somewhat different manner from pathologist to pathologist, despite attempts to utilise identical criteria. The difficulties associated with the use of morphological features as risk factors have been discussed by other authors (Gilchrist *et al.*, 1985).

Considering the evidence correlating EIC with residual tumour burden, it is not surprising that resection margins in EIC + patients appear less commonly to be adequate. The confounding of EIC and resection margin status as competing risk factors has been alluded to by the Harvard group (Recht *et al.*, 1986). The exclusion of the 10% of patients having positive margins from breast-conserving therapy may account for the failure of the pathologists of the National Surgical Adjuvant Breast Project to identify EIC as a risk factor for local failure (Fisher *et al.*, 1986). Harris *et al.* (1985) have proposed a strategy of re-excision in patients with EIC, in the hopes of reducing local recurrence rates by achieving more generous margins. Our retrospective analysis of resection margins, however, suggests that local recurrence risk may be inherently high for EIC + patients, despite apparently adequate excision. This merits more careful prospective evaluation.

The most important finding of our study was the notion that EIC may have limited importance as a risk factor in post-menopausal patients. We found that EIC is distinctly more prevalent in patients younger than 50, and that the extreme form of EIC is seen predominantly in women younger than 40 (Table III). The latter correlation may be at least partially responsible for the higher breast recurrence risk observed in this young age group (Calle *et al.*, 1986; Recht *et al.*, 1988; Kurtz *et al.*, 1988). An association between extent of intraductal component and young age has been noted previously by us (Jacquemier *et al.*, 1985) as well as by other authors (Fisher *et al.*, 1975; Recht *et al.*, 1988; Migliori, 1982).

Moreover, our data suggest that EIC is not only less prevalent in older patients, but that its presence has little influence on local recurrence risk after the menopause (Table IV). Similarly, Bartelink *et al.* (1988) found a positive association between 25% or more DCIS and local recurrence for patients younger than 50, but none for older patients . In addition, the Harvard data show no influence of EIC for patients older than 65, but suggest that this feature is predictive of local failure for the age groups less than 35, 35–50 and 51–65. These observations raise the possibility that the reliability of EIC as a marker of residual cancer decreases with increasing age, or that residual tumour responds differently to treatment as a function of age.

Aside from its correlation with local recurrence risk, EIC

**Table IV** Influence of extensive intraductal component (EIC) on local recurrence in the breast for 496 infiltrating ductal cancers, according to menopausal status

| | *Crude breast recurrence rate* | |
|---|---|---|
| Premenopausal ($n$ = 231) | | |
| EIC + | 17/65 (26%) | $P<0.001$ |
| EIC − | 13/166 (8%) | |
| Post-menopausal ($n$ = 265) | | |
| EIC + | 5/41 (12%) | $P>0.8$ |
| EIC − | 26/224 (12%) | |

did not appear to have any other prognostic significance in our series. There was no significant difference in overall survival between patients with or without EIC (Figure 2), a similar observation having been made by Boyages et al. (1989). Additionally, as far as could be judged by rather limited follow-up, the clinical behaviour of local recurrences in both groups of patients appeared to be similar, in that both the median time to local failure and short-term survival after relapse were the same for EIC + and EIC − patients.

In summary, our analysis confirms the Harvard data, as well as the subsequent studies of Bartelink et al. (1988), Lindley et al. (1989) and Fourquet et al. (1989), demonstrating a significant association between EIC and the risk of failure in the breast after limited tumour excision and radiotherapy. However, the usefulness of EIC as a risk factor may be limited particularly by the age dependence of this feature. It is possible that EIC reflects a form of local tumour growth which depends on high circulating estrogen levels for its propagation, and that this feature becomes both less common and biologically less significant in post-menopausal patients.

In contrast to the earliest reports (Schnitt et al., 1984), it is now apparent that local failure is observed in substantial numbers of conservatively treated patients whose tumours had not demonstrated EIC, so that recurrence risk cannot be adequately assessed by this single factor. Given the increasing popularity of breast-conserving treatment, continued clinical investigation in this area is certainly justified. The relative importance of EIC and resection margins as competing markers of residual tumour burden should be evaluated prospectively. For a given tumour burden, moreover, the risk of local failure after potentially curative radiotherapy may also depend upon histopathologic characteristics of the primary lesion, making it likely that other factors in addition to EIC and resection margins will prove to be important. Examples of features identified thus far include histologic grade (Lindley et al., 1989; Kurtz et al., 1990), prominent lymphocytic stromal resection (Lindley et al., 1989; Kurtz et al., 1990, tumour necrosis (Lindley et al., 1989) and vascular invasion (Fisher et al., 1986; Fourquet et al., 1989). Assessment of the relative value of these morphological features, as well as of various biological measures of tumour aggressiveness, will require additional study.

### References

AMERICAN JOINT COMMITTEE (1983). *Manual for Staging of Cancer*, p. 127. J.B. Lippincott: Philadelphia.

BARTELINK, H., BORGER, J.H., VAN DONGEN, J.A. & PETERSE, J.L. (1988). The impact of tumor size and histology on local control after breast conserving therapy. *Radiother. Oncol.*, **11**, 297.

BOYAGES, J., RECHT, A., CONNOLLY, J. & 4 others (1989). Factors associated with local recurrence as a first site of failure following the conservative treatment of early breast cancer. In *Adjuvant Therapy of Primary Breast Cancer*, Senn, H.-J. (ed.) p. 92. Springer-Verlag: Heidelberg.

CALLE, R., VILCOQ, J.R., ZAFRANI, B., VIELH, P. & FOURQUET, A. (1986). Local control and survival of breast cancer treated by limited surgery followed by irradiation. *Int. J. Radiat. Oncol. Biol. Phys.*, **12**, 873.

FISHER, E.R., GREGORIO, R.M., FISHER, B. et al. (1975). The pathology of invasive breast cancer. A syllabus derived from findings of the National Surgical Adjuvant Breast Project (protocol no. 4). *Cancer*, **36**, 1.

FISHER, E.R., SASS, R., FISHER, B. et al. (1986). Pathologic findings from the National Surgical Adjuvant Breast Project (protocol 6). II. Relation of local breast recurrence to multicentricity. *Cancer*, **57**, 1717.

FOURQUET, A., CAMPANA, F., ZAFRANI, B. et al. (1989). Prognostic factors of breast recurrence in the conservative management of early breast cancer: a 25-year follow-up. *Int. J. Radiat. Oncol. Biol. Phys.*, **17**, 719.

GILCHRIST, K.W., KALISH, L., GOULD, V.E. et al. (1985). Interobserver reproducibility of histopathological features in stage II breast cancer. *Breast Cancer Res. Treat.*, **5**, 3.

HARRIS, J.R., CONNOLLY, J.L., SCHNITT, S. et al. (1985). The use of pathologic features in selecting the extent of surgical resection necessary for breast cancer patients treated by primary radiation therapy. *Ann. Surg.*, **201**, 164.

HOLLAND, R., CONNOLLY, J., GELMAN, R. et al. (1990). The presence of an extensive intraductal component (EIC) following a limited excision correlates with prominent residual disease in the remainder of the breast. *J. Clin. Oncol.* (in the press).

JACQUEMIER, J., SERADOUR, B., HASSOUN, J. & PIANA, L. (1985). Special morphologic features of invasive mammary carcinomas in women under 40 years of age. *Breast Dis.*, **1**, 119.

KAPLAN, E.L. & MEIER, P. (1958). Nonparametric estimation from incomplete observations. *J. Am. Stat. Assoc.*, **53**, 457.

KURTZ, J.M., JACQUEMIER, J., AMALRIC, R. et al. (1990). Risk factors for breast recurrence in pre- and post-menopausal patients with ductal cancers treated by conservation therapy. *Cancer* (in the press).

KURTZ, J.M., JACQUEMIER, J., TORHORST, J. et al. (1989). Conservation therapy for breast cancers other than infiltrating ductal carcinoma. *Cancer*, **63**, 1630.

KURTZ, J.M., SPITALIER, J.M., AMALRIC, R. et al. (1988). Mammary recurrence in women younger than forty. *Int. J. Radiat. Oncol. Biol. Phys.*, **15**, 271.

LINDLEY, R., BULMAN, A., PARSONS, P. et al. (1989). Histologic features predictive of an increased risk of early local recurrence after treatment of breast cancer by local tumor excision and radical radiotherapy. *Surgery*, **105**, 13.

MIGLIORI, E. (1982). Variations according to patient age in certain histopathological parameters in cancer of the breast. *Tumori*, **68**, 403.

PETO, R., PIKE, M.C., ARMITAGE, P. et al. (1977). Design and analysis of randomised clinical trials requiring prolonged observation of each patient. *Br. J. Cancer*, **35**, 1.

RAPIN, V., CONTESSO, G., MOURIESSE, H. et al. (1988). Medullary breast cancer. A re-evaluation of 95 cases of breast cancer with inflammatory stroma. *Cancer*, **61**, 2503.

RECHT, A., CONNOLLY, J.L., SCHNITT, S.J. et al. (1986). Conservative surgery and radiation therapy for early breast cancer: results, controversies, and unsolved problems. *Semin. Oncol.*, **13**, 434.

RECHT, A., CONNOLLY, J.L., SCHNITT, S.J. et al. (1988). The effect of young age on tumor recurrence in the treated breast after conservative surgery and radiotherapy. *Int. J. Radiat. Oncol. Biol. Phys.*, **14**, 3.

SCHNITT, S.J., CONNOLLY, J.L., HARRIS, J.R. et al. (1984). Pathologic predictors of early local recurrence in stage I and II breast cancer treated by primary radiation therapy. *Cancer*, **53**, 1049.

SCHNITT, S.J., CONNOLLY, J.L., KHETTRY, U. et al. (1987). Pathologic findings on re-excision of the primary site in breast cancer patients considered for treatment by primary radiation therapy. *Cancer*, **59**, 675.

WORLD HEALTH ORGANIZATION (1982). Histologic typing of breast tumors. *Am. J. Clin. Pathol.*, **78**, 806.


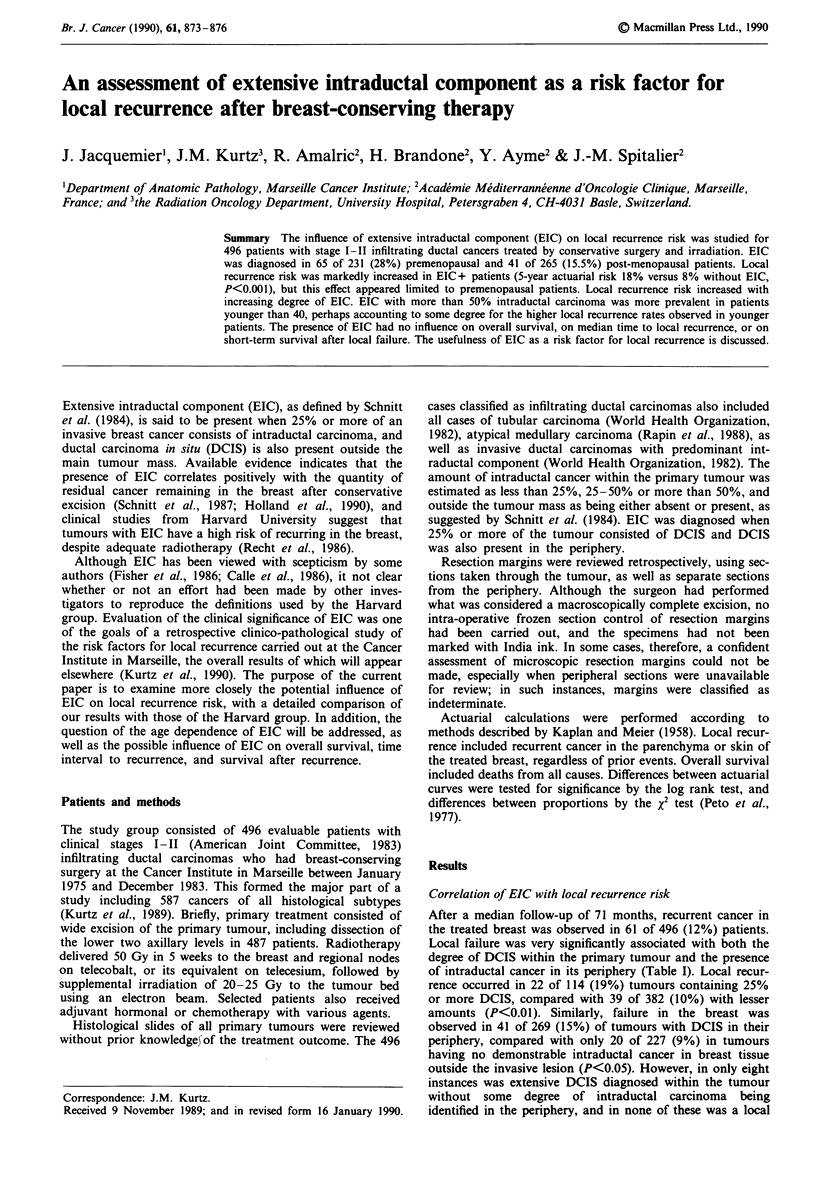

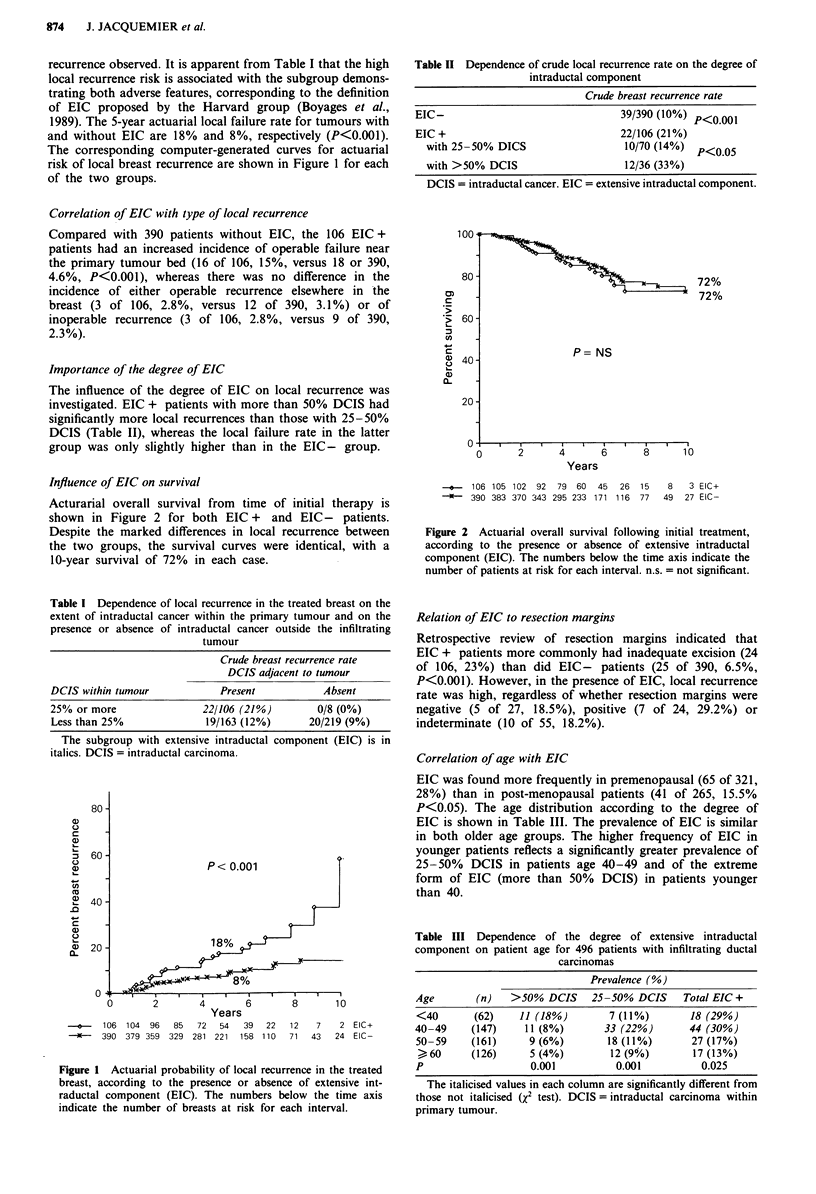

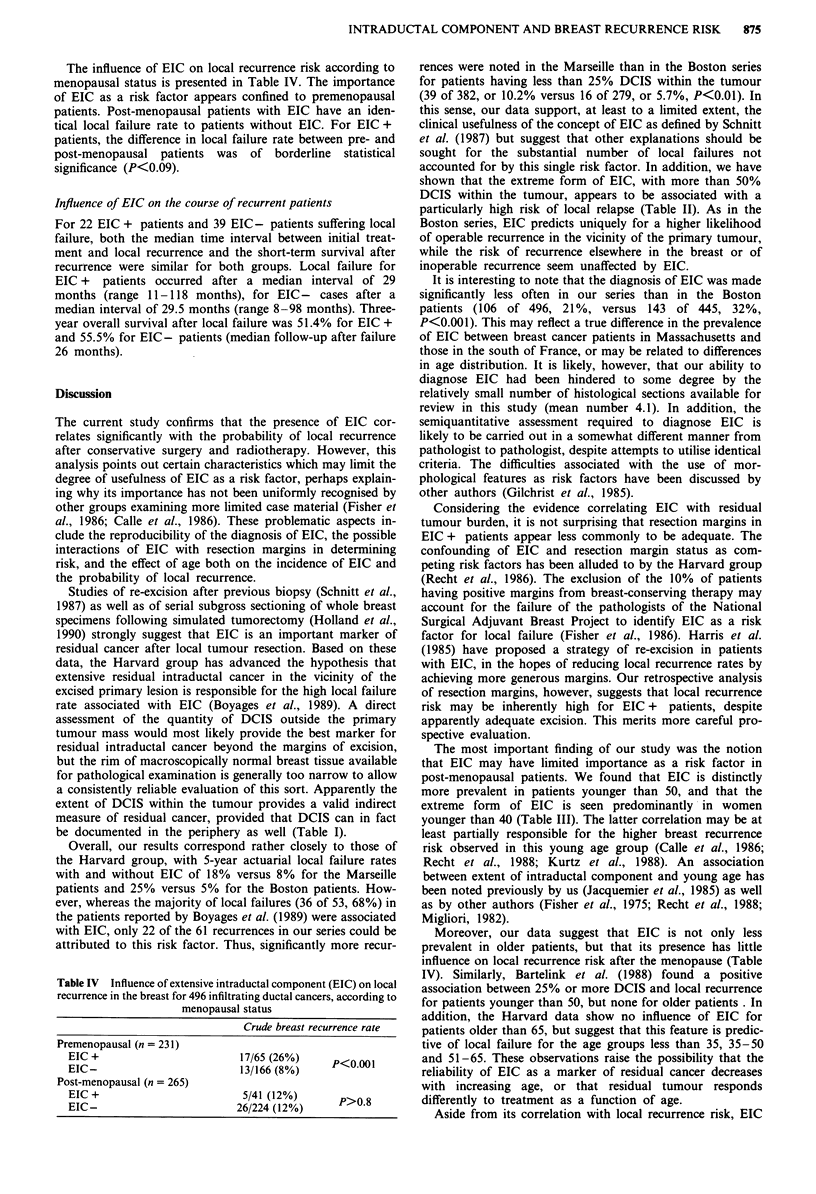

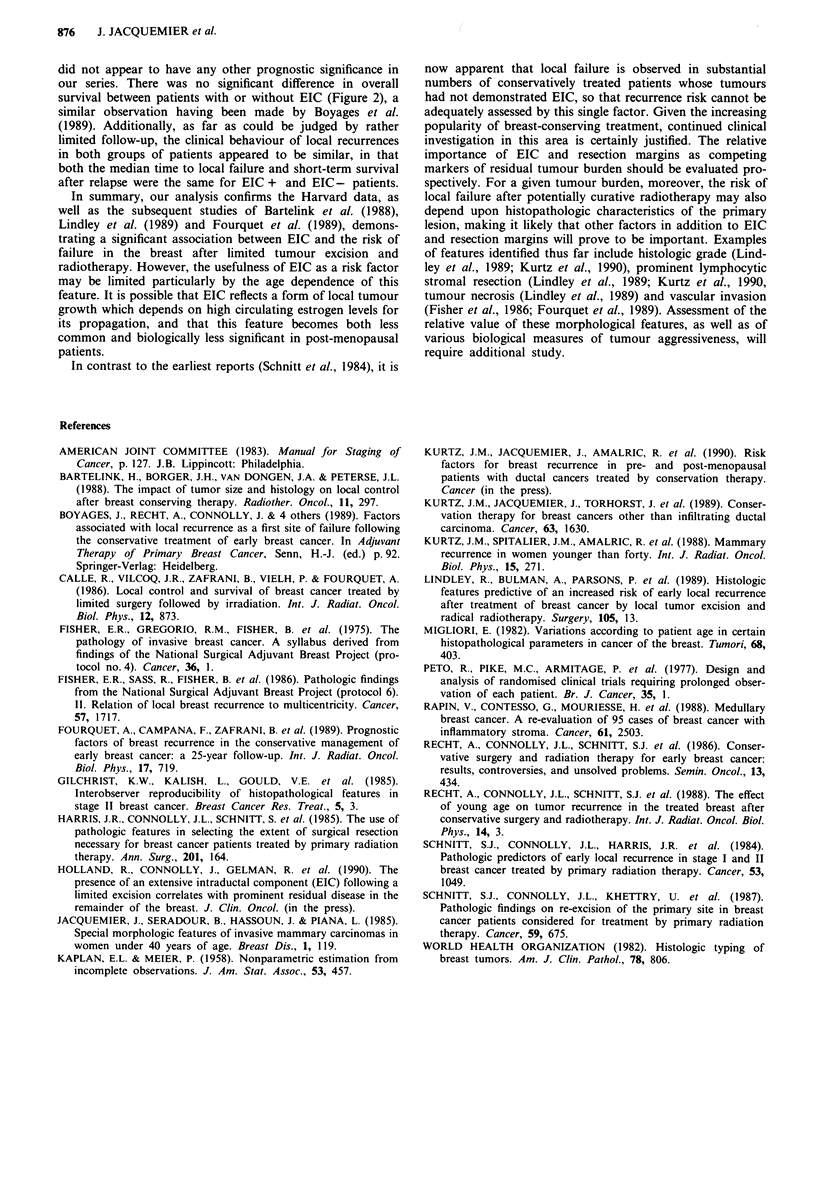


## References

[OCR_00545] Bartelink H., Borger J. H., van Dongen J. A., Peterse J. L. (1988). The impact of tumor size and histology on local control after breast-conserving therapy.. Radiother Oncol.

[OCR_00557] Calle R., Vilcoq J. R., Zafrani B., Vielh P., Fourquet A. (1986). Local control and survival of breast cancer treated by limited surgery followed by irradiation.. Int J Radiat Oncol Biol Phys.

[OCR_00563] Fisher E. R., Gregorio R. M., Fisher B., Redmond C., Vellios F., Sommers S. C. (1975). The pathology of invasive breast cancer. A syllabus derived from findings of the National Surgical Adjuvant Breast Project (protocol no. 4).. Cancer.

[OCR_00569] Fisher E. R., Sass R., Fisher B., Gregorio R., Brown R., Wickerham L. (1986). Pathologic findings from the National Surgical Adjuvant Breast Project (protocol 6). II. Relation of local breast recurrence to multicentricity.. Cancer.

[OCR_00575] Fourquet A., Campana F., Zafrani B., Mosseri V., Vielh P., Durand J. C., Vilcoq J. R. (1989). Prognostic factors of breast recurrence in the conservative management of early breast cancer: a 25-year follow-up.. Int J Radiat Oncol Biol Phys.

[OCR_00581] Gilchrist K. W., Kalish L., Gould V. E., Hirschl S., Imbriglia J. E., Levy W. M., Patchefsky A. S., Penner D. W., Pickren J., Roth J. A. (1985). Interobserver reproducibility of histopathological features in stage II breast cancer. An ECOG study.. Breast Cancer Res Treat.

[OCR_00586] Harris J. R., Connolly J. L., Schnitt S. J., Cady B., Love S., Osteen R. T., Patterson W. B., Shirley R., Hellman S., Cohen R. B. (1985). The use of pathologic features in selecting the extent of surgical resection necessary for breast cancer patients treated by primary radiation therapy.. Ann Surg.

[OCR_00613] Kurtz J. M., Jacquemier J., Torhorst J., Spitalier J. M., Amalric R., Hünig R., Walther E., Harder F., Almendral A., Brandone H. (1989). Conservation therapy for breast cancers other than infiltrating ductal carcinoma.. Cancer.

[OCR_00618] Kurtz J. M., Spitalier J. M., Amalric R., Brandone H., Ayme Y., Bressac C., Hans D. (1988). Mammary recurrences in women younger than forty.. Int J Radiat Oncol Biol Phys.

[OCR_00623] Lindley R., Bulman A., Parsons P., Phillips R., Henry K., Ellis H. (1989). Histologic features predictive of an increased risk of early local recurrence after treatment of breast cancer by local tumor excision and radical radiotherapy.. Surgery.

[OCR_00629] Migliori E. (1982). Variations according to patient-age in certain histopathological parameters in cancer of the breast.. Tumori.

[OCR_00634] Peto R., Pike M. C., Armitage P., Breslow N. E., Cox D. R., Howard S. V., Mantel N., McPherson K., Peto J., Smith P. G. (1977). Design and analysis of randomized clinical trials requiring prolonged observation of each patient. II. analysis and examples.. Br J Cancer.

[OCR_00639] Rapin V., Contesso G., Mouriesse H., Bertin F., Lacombe M. J., Piekarski J. D., Travagli J. P., Gadenne C., Friedman S. (1988). Medullary breast carcinoma. A reevaluation of 95 cases of breast cancer with inflammatory stroma.. Cancer.

[OCR_00644] Recht A., Connolly J. L., Schnitt S. J., Cady B., Love S., Osteen R. T., Patterson W. B., Shirley R., Silen W., Come S. (1986). Conservative surgery and radiation therapy for early breast cancer: results, controversies, and unsolved problems.. Semin Oncol.

[OCR_00650] Recht A., Connolly J. L., Schnitt S. J., Silver B., Rose M. A., Love S., Harris J. R. (1988). The effect of young age on tumor recurrence in the treated breast after conservative surgery and radiotherapy.. Int J Radiat Oncol Biol Phys.

[OCR_00656] Schnitt S. J., Connolly J. L., Harris J. R., Hellman S., Cohen R. B. (1984). Pathologic predictors of early local recurrence in Stage I and II breast cancer treated by primary radiation therapy.. Cancer.

[OCR_00662] Schnitt S. J., Connolly J. L., Khettry U., Mazoujian G., Brenner M., Silver B., Recht A., Beadle G., Harris J. R. (1987). Pathologic findings on re-excision of the primary site in breast cancer patients considered for treatment by primary radiation therapy.. Cancer.

